# Therapeutic effect of ibrutinib, a selective Bruton’s tyrosine kinase inhibitor, in orbital fibroblasts from patients with Graves’ orbitopathy

**DOI:** 10.1371/journal.pone.0279060

**Published:** 2022-12-15

**Authors:** Hyun Young Park, Min Kyung Chae, JaeSang Ko, Don O. Kikkawa, Sun Young Jang, Jin Sook Yoon

**Affiliations:** 1 Department of Ophthalmology, Severance Hospital, Institute of Vision Research, Yonsei University College of Medicine, Seoul, Republic of Korea; 2 Siloam Eye Hospital, Seoul, Republic of Korea; 3 Division of Oculofacial Plastic and Reconstructive Surgery, Department of Ophthalmology, University of California San Diego, La Jolla, California, United States of America; 4 Department of Ophthalmology, Soonchunhyang University Bucheon Hospital, Soonchunhyang University College of Medicine, Bucheon, Korea; University of Iowa, UNITED STATES

## Abstract

**Purpose:**

Bruton’s tyrosine kinase (BTK) is an essential protein in B-cell antigen receptor (BCR) signaling pathway and is known to be related to pathogenetic effect on B-cell related malignancies and various autoimmune diseases. In this study, we investigated the therapeutic effect of ibrutinib, an orally bioavailable BTK inhibitor in the pathogenesis of Graves’ orbitopathy (GO) in *in vitro* model.

**Methods:**

Expression of BTK in orbital tissues from GO and normal control subjects were evaluated by real-time polymerase chain reaction (PCR). Primary cultured orbital fibroblasts from each subject were exposed to ibrutinib and stimulated with interleukin (IL)-1β or insulin like growth factor (IGF)-1. Production of inflammatory cytokines was evaluated by real time PCR and enzyme-linked immunosorbent assays (ELISA). The downstream transcription factors were also determined by western blot assays.

**Results:**

The expression of BTK in GO tissues were significantly higher than in healthy controls. After stimulation of GO orbital fibroblasts with IL-1β or IGF-1, BTK mRNA and phosphorylated (p)- BTK protein expression was also enhanced. Ibrutinib reduced the expression of BTK mRNA and proteins of p-BTK, and inhibited the IL-1β- and IGF-1-induced production of proinflammatory cytokines including IL-6, IL-8 and COX-2 in both GO and normal cells. Ibrutinib also significantly attenuated phosphorylation of Akt, p38, c-Jun N-terminal kinase (JNK), extracellular signal-regulated kinase (ERK), and nuclear factor kappa-light-chain-enhancer of activated B-cells (NF-κB) in IL-1β stimulated GO cells and Akt, JNK, and NF-κB in IL-1ß stimulated normal cells.

**Conclusions:**

BTK expression is enhanced in GO tissue and orbital fibroblasts. Ibrutinib, a BTK inhibitor suppresses proinflammatory cytokine production as well as phosphorylation of Akt and NF-κB protein. Our results suggest the potential role of BTK in GO inflammatory pathogenesis and possibility of a novel therapeutic target of GO.

## Introduction

Graves’ orbitopathy (GO) is a pathologic manifestation of Graves’ disease, a rare autoimmune inflammatory disorder. This autoimmune condition is caused by thyrotropin receptor and insulin-like growth factor-1 (IGF-1) receptor autoantibodies triggering diffuse autoreactive T-cell and B-cell infiltration, leading to orbital inflammation [1–3]. Orbital fibroblasts, stimulated by mononuclear cells, have been implicated in pathogenesis of GO [4, 5]. Proliferation and differentiation of activated orbital fibroblasts induces extracellular matrix deposition and adipogenesis [6, 7]. This massive soft tissue enlargement and remodeling in GO results in periorbital swelling, proptosis, pain, double vision, and possible vision threatening complications. As inflammation is one of the main manifestations in GO, glucocorticoids are traditionally used as first line treatment in the acute inflammatory phase [8], however, due to side effects and treatment failures, new agents targeting the autoimmune process at the molecular level have been suggested [9–11].

Bruton’s tyrosine kinase (BTK), a tyrosine kinase that is encoded by *BTK* gene, plays an essential role in B-cell differentiation, proliferation, and survival, which is critical in the downstream regulation of the B-cell antigen receptor (BCR) signaling pathway [12–14]. Furthermore, BTK is also involved in other signaling pathways including B-cell-activating factor receptors, chemokine receptors, Toll-like receptors (TLR), and Fc receptor signaling in B-cells and myeloid lineage cells, such as macrophages and dendritic cells [15–18]. In the context of its extensive role in several immunologic pathways, potential therapeutic effects of targeting BTK is currently being explored. Initially designed as a treatment in the rheumatoid arthritis mouse model [19], ibrutinib, an orally bioavailable selective BTK inhibitor, has been approved for the treatment for the various B-cell malignancies and chronic graft-versus-host disease [20–24]. Furthermore, ibrutinib reverses the inflammatory response in other disorders, such as autoimmune arthritis and systemic lupus erythematosus in animal model [25–27].

In view of the fundamental role of BTK in autoimmune disease, this study was conducted to characterize the role of BTK in an *in vitro* model of GO. We investigated the expression level of BTK in GO tissues compared to that in normal orbital tissues and evaluated the anti-inflammatory effect of ibrutinib in primary cultured orbital fibroblasts.

## Materials and methods

### Reagents

The source of the reagents were as follows: ibrutinib (Selleckchem chemicals, Houston, TX, US); fetal bovine serum (FBS) (Gibco, Waltham, MA, USA); Dulbecco’s modified Eagle’s medium (DMEM), phosphate buffer saline (PBS), penicillin and gentamicin (Welgene, Gyeongsangbuk-do, Gyeongsan-si, South Korea); 3-(4,5-dimethyl-thiazol-2-yl)-2,5-diphenyl-tetrazolium bromide (MTT) assay (Sigma-Aldrich, Inc, St. Louis, MO, USA); recombinant human interleukin (IL)-1β, IGF-1, and the enzyme-linked immunosorbent assay (ELISA) kits for IL-6 and IL-8 (R&D systems, Minneapolis, MN, USA); antibodies for phosphorylated (p)- and total (t)- Akt, p-p38 mitogen-activated protein kinase (MAPK), t-p38, p-c-Jun N-terminal kinase (JNK), t-JNK, p-extracellular signal-regulated kinase (ERK), t-ERK, p-nuclear factor κ-light-chain-enhancer of activated B-cells (NF-κB), t-NF-κB, and p-BTK T223 (Cell Signaling Technology, Danvers, MA, US); t-BTK (Abcam, Cambridge, UK) antibody; β-actin (Santa Cruz Biotechnology, Santa Cruz, CA, USA) antibody; and p-BTK Y551 antibody (BD Biosciences, San Jose, CA, USA).

### Cell culture

Orbital adipose and connective tissue specimens were obtained during orbital decompression surgery in 15 patients with GO. At the time of the surgery, all patients were in a stable euthyroid state and were not treated with radiotherapy or steroid treatment for at least three months prior to the time of surgery. All healthy controls were free from thyroid disease and their tissue specimens were harvested during upper or lower lid blepharoplasty (n = 14, S1 Table). Written informed consent was obtained from all patients. The study protocol was approved by the institutional review board of Soonchunhyang Hospital, Soonchunhyang University College of Medicine (2020-04-006) and Severance Hospital, Yonsei University College of Medicine (4-2022-0272), and was conducted in accordance with tenets of the Declaration of Helsinki. Orbital fibroblast cultures were performed as previously described [28]. Concisely, tissue explants were minced and placed in 1:1 DMEM:F12 medium containing 20% FBS, penicillin, and gentamicin. Then, monolayers were passaged serially with trypsin/EDTA after the cell growth was confirmed. The cell strains were stored in liquid nitrogen and cells between the third and seventh passage were used.

### Quantitative real-time PCR

To assess BTK gene level in each orbital sample, a tissue homogenizer (Precellys 24; Bertin Instruments, Montigny-le-Bretonneux, France) and a Precellys lysing kit (Bertin Instruments) were used for orbital tissue homogenization. Total RNA was extracted from orbital fibroblasts with TriZol agent (Invitrogen, Carlsbad, CA, USA). cDNA was synthesized from 1 μg of RNA and amplified with TaqMan universal polymerase chain reaction (PCR) master mix (Applied Biosystems, Foster City, CA, USA) in an ABI 7300 real-time PCR thermocycler (Applied Biosystems, Carlsbad, CA, USA). Primer sequences for each gene was as follows: IL-6 forward 5’-AGTTCCTGC AGAAAAAGGCAAAG-3’ and reverse 5’- CATTTGCCGAAGAGCCCTCA-3’; IL-8 forward 5’-CAATCCTAGTTTGATACTCCC-3’ and reverse 5’- AATTACTAATATTGACTGTGGAG-3’; COX-2 forward 5’- CCCTTGGGTGTCAAAGGTAA-3’ and reverse 5’- GCCCTCGCTTATGATCTGTC-3’; glyceraldehyde 3-phosphate dehydrogenase (GAPDH) forward 5’-ATGGGGAAGGTGAAGGTCG-3’ and reverse 5’- GGGGTCATTGATGGCAACAATA-3’; BTK forward 5’-GGCTCCAAATTTCCAGTCCG-3’ and reverse 5’-AACCCCAAAAGCCCAAATGTC-3’; hyaluronan (HA) synthase 1 forward 5’-GGAATAACCTCTTGCAGCAGTTTC-3’ and reverse 5’-GCCGGTCATCCCCAAAAG-3’; HA synthase (HAS) 2 forward 5’-TCGCAACACGTAACGCAAT-3’ and reverse 5’-ACTTCTCTTTTTCCACCCCATTT-3’; HAS3 forward 5’-AACAAGTACGACTCATGGATTTCCT-3’ and reverse 5’-GCCCGCTCCACGTTGA-3’; monocyte chemoattractant protein 1 (MCP-1) forward 5’-TCCCAAAGAAGCTGTGATCTTCA-3’ and reverse 5’-TTTGCTTGTCCAGGTGGTCC-3’. To normalize the results, GAPDH expression was used. The results were expressed as relative fold changes in the threshold cycle (Ct) value using 2^- ΔΔ Ct^ method.

### Cell viability

To determine the non-cytotoxic concentration of ibrutinib on orbital fibroblasts, orbital fibroblasts of GO patients were seeded into 24-well culture plates (1 x 10^5^ cells/well) and treated with serial concentrations of ibrutinib (control, 0.5, 1, 2, 5, and 10 μM) for 16, 24, and 48 hours. After the exposure, cells were washed and incubated with 5 mg/mL MTT solution at 37°C for 3 hours. Then, ice-cold isopropanol was applied for solubilization, and absorbance of the dye was measured at 560 nm with background subtraction at 630 nm using a microplate reader (EL 340 Bio Kinetics Reader; Bio-Teck Instruments, Winooski, VT, USA). Cell viability was expressed as a percentage relative to untreated control cells.

### Western blotting assay

To examine the inhibitory effect of ibrutinib on BTK phosphorylation, orbital fibroblasts were treated with ibrutinib (1 μM) for 2 and 24 hours. Orbital fibroblasts were also pre-treated with ibrutinib (1 μM) for 24 hours and were then stimulated with IL-1β (10 ng/mL) or IGF-1 (10 ng/mL) for 1 hour to assess the intracellular signaling pathway. Then the orbital fibroblasts, after exposure to the varying study conditions, were washed with PBS and lysed with cell lysis buffer as described previously [28]. The centrifuged cell lysates were boiled and resolved by 10% SDS-polyacrylamide gel electrophoresis (SDS-PAGE). Proteins were then transferred to nitrocellulose membranes (Millipore Corp., Billerica, MA, USA) and treated with antibodies at 4°C overnight. Immunoreactive bands were detected with horseradish peroxidase-conjugated secondary antibody and were visualized by chemiluminescence (Amersham Pharmacia Biotech, Inc., Piscataway, NJ, USA). The relative intensity of total protein in each band was quantified by densitometry and normalized to that of β-actin, as a positive control. The activated level of each protein was determined by the fraction of phosphorylated protein level compared to total protein level. Full length gels representative of Western blot analysis were provided in S1 Fig.

### ELISA

Cell supernatants were collected from orbital fibroblasts of GO and normal patients, and the levels of IL-6 and IL-8 were quantified using a commercially available ELISA kit. The absorbance was measured at 450 nm and the percentage of binding was calculated for each sample, and a standard binding curve was generated to determine the concentrations. Average value of three assays were used for statistical analysis.

### Statistical analysis

At least three strains from three different individuals were used in the experiments. Each experiment per sample was performed triplicate. For nonparametric data, Mann-Whitney U-test and Kruskal Wallis test were used and for data without normal distribution, Kolmogorov-Smirnov test was used. SPSS Statistics 22 (IBM, Armonk, NY, USA) was used for statistical analysis and p-value less than 0.05 were considered statistically significant.

## Results

### Increased expression of BTK in GO tissues

**Fig 1:**
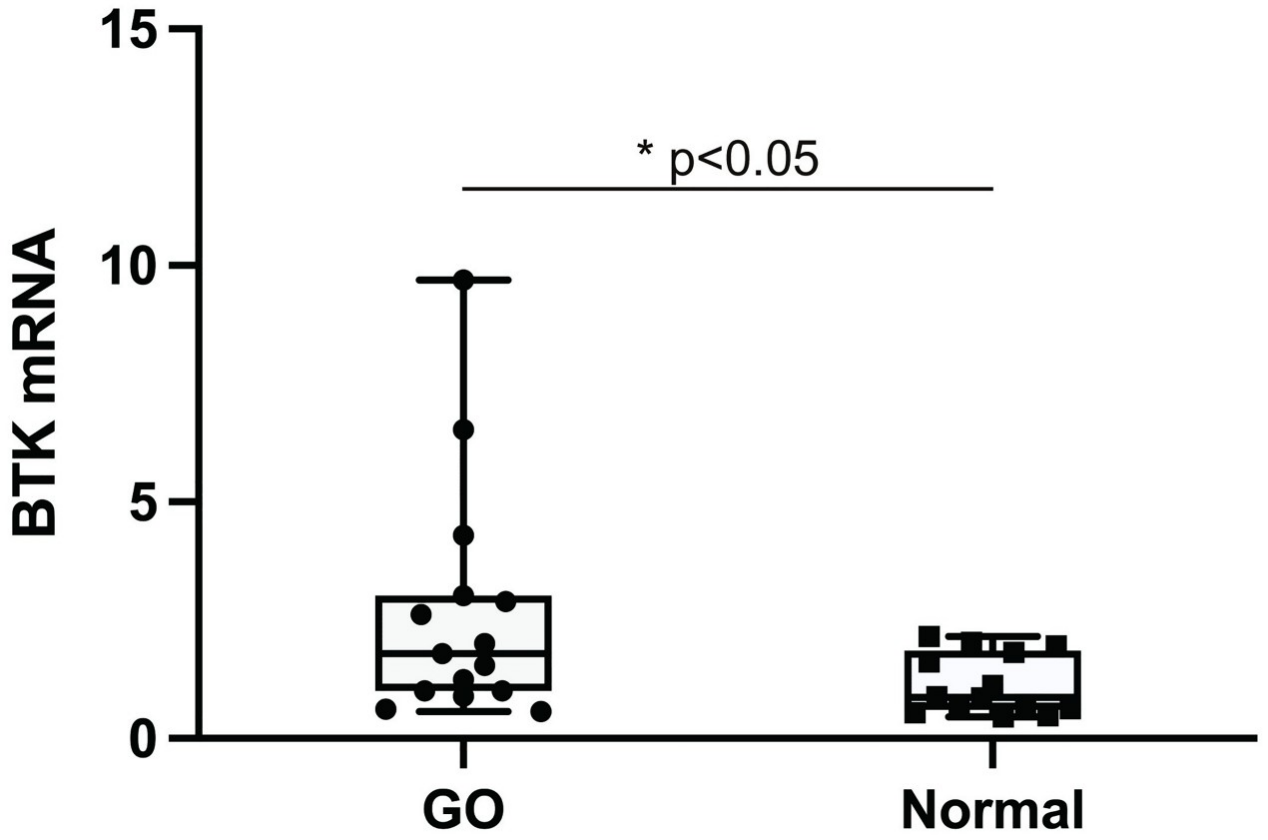
Expression of Bruton’s tyrosine kinase (BTK) mRNA in orbital tissues. Orbital tissues from individuals with Graves’ orbitopathy (GO) (n = 15) and normal controls (n = 14) were used to evaluate the transcription level of BTK mRNA using real-time PCR. The results are presented as the median and interquartile range. (*p<0.05 versus normal control tissues).

mRNA was extracted from orbital tissues of each GO subjects and normal controls and BTK transcription levels were compared between GO and normal tissues using RT-PCR (Fig 1). The RT-PCR result showed that the basal BTK expression level was significantly higher in GO tissues (n = 15) compared to normal tissues (n = 14).

**Fig 2:**
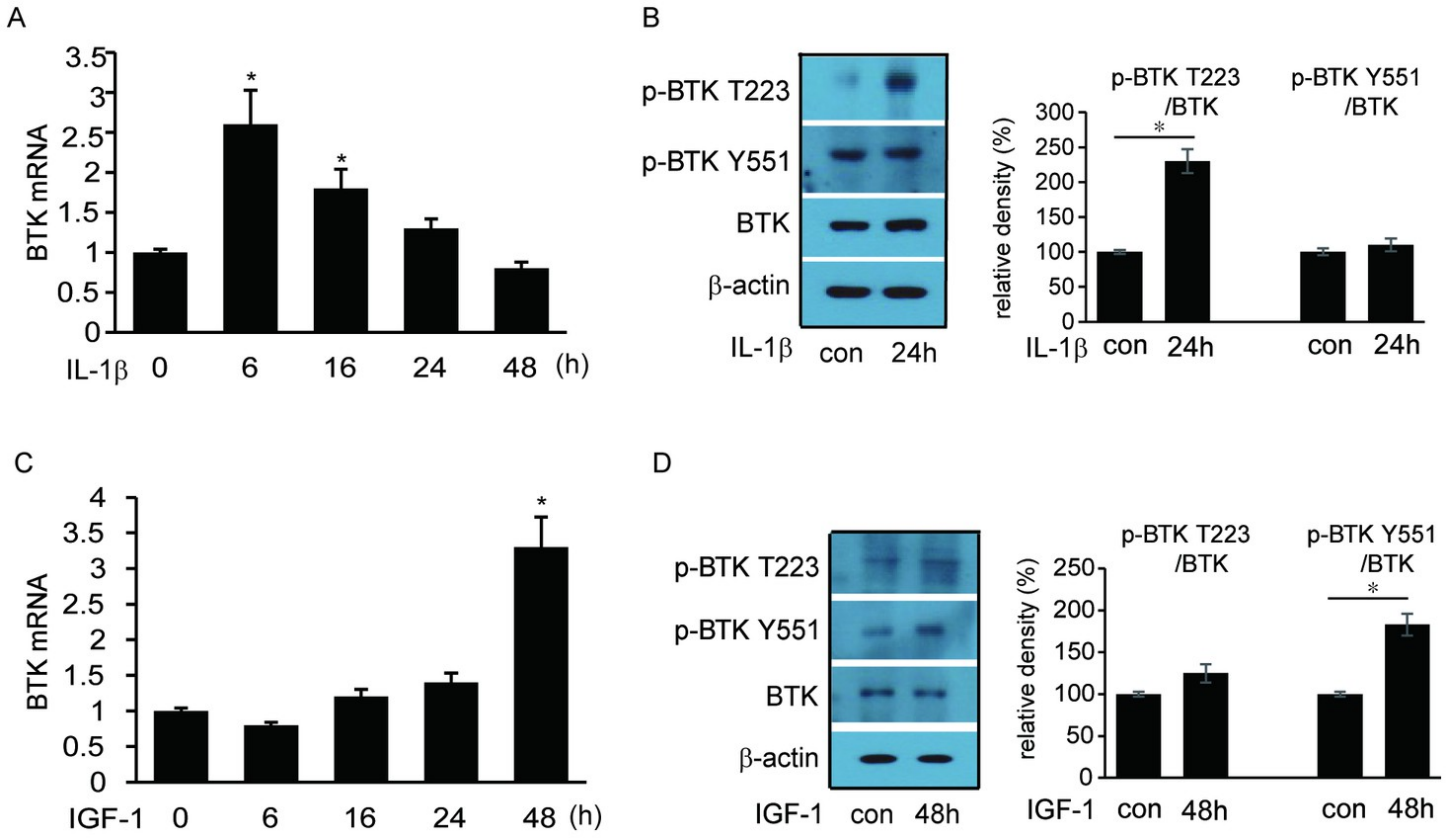
Effect of IL-1β and IGF-1 on Bruton’s tyrosine kinase (BTK) mRNA and protein expression in Graves’ orbitopathy (GO) orbital fibroblasts. Transcription and phosphorylation of Bruton’s tyrosine kinase (BTK) in Graves’ orbitopathy (GO) fibroblast in response to IL-1β (10 ng/mL) and IGF-1 (10ng/mL) exposure for increasing length of time (0–48 hours for both IL-1β and IGF-1) were evaluated. Total RNA (1 μg) extracted from each sample was reverse transcribed and amplified by real-time PCR. (A) Orbital fibroblasts obtained from GO patients were treated with IL-1β. BTK mRNA expression was increased after IL-1β treatment, which peaked 6 hours after the treatment (*p<0.05). (B) Western blot assays were performed to evaluate the expression levels of phosphorylated BTK level. The relative density of phosphorylated BTK at T223 domain was higher after 24-hour IL-1β exposure, whereas the phosphorylated level of Y551 did not show significant difference (*p<0.05). (C) Orbital fibroblasts obtained from GO tissues were stimulated with IGF-1 and BTK mRNA expression level was increased 48 hours after the treatment (*p<0.05). (D) The relative density of phosphorylated BTK at Y551 domain was significantly increased after treatment of IGF-1 for 48 hours, whereas T223 domain phosphorylation was not (*p<0.05).

BTK transcript levels were evaluated after stimulation of IL-1β (10 ng/mL) and IGF-1(10 ng/mL) for various time periods. BTK mRNA expression increased after IL-1β and IGF-1 treatment, which peaked at 6 hours of IL-1β and 48 hours of IGF-1 treatment (Fig 2A and 2C). BTK transcripts phosphorylated at the T223 domain site significantly increased after 24 hour-treatment with IL-1β. (Fig 2B) After 48 hours of IGF-1 treatment, BTK transcripts phosphorylated at the Y551 domain site increased (Fig 2D).

### Viability of orbital fibroblasts after ibrutinib treatment

**Fig 3:**
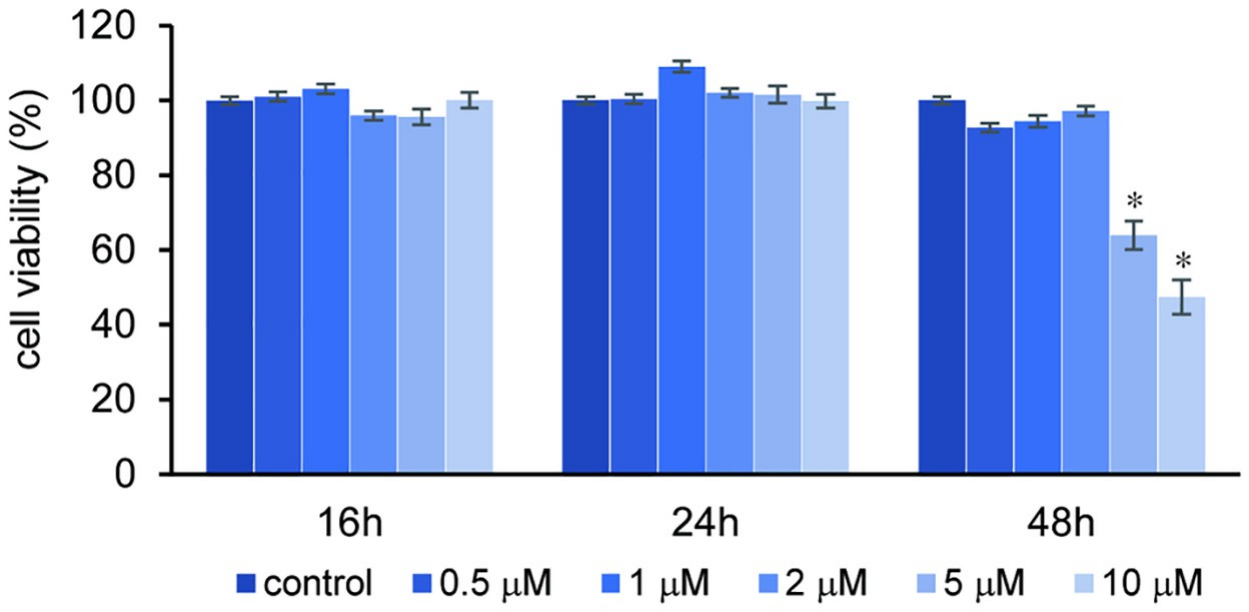
Effect of ibrutinib on the viability of orbital fibroblasts. Orbital fibroblasts from Graves’ orbitopathy subjects were seeded in 24-well culture plates and treated with variable concentration (0–10 μM) of ibrutinib for 16, 24, and 48 hours. A concentration of 5 μM and 10 μM of ibrutinib for 48 hours significantly inhibited cell viability (*p<0.05 versus control). MTT assays were performed in duplicate using cells from three different donors. Results are expressed as percentages of untreated control values and are presented as means ± standard deviation.

To identify the non-toxic concentrations of ibrutinib in orbital fibroblasts, an MTT assay was conducted. Orbital fibroblast from subjects with GO were treated with ibrutinib 0.5–10 μM for 16, 24, and 48 hours. Treatment with ibrutinib 0–10 μM range revealed cell viability of more than 95% for 16 and 24 hours (Fig 3). Treatment of ibrutinib for 48 hours showed cell viability of more than 90%, at a dose of not more than 2μM. Based on this result, the maximal nontoxic concentration of ibrutinib was determined to be 10 μM for 24 hours in orbital fibroblasts and 1μM was applied for the subsequent assays.

### Effect of Ibrutinib on proinflammatory cytokine expression and HAS expression

**Fig 4:**
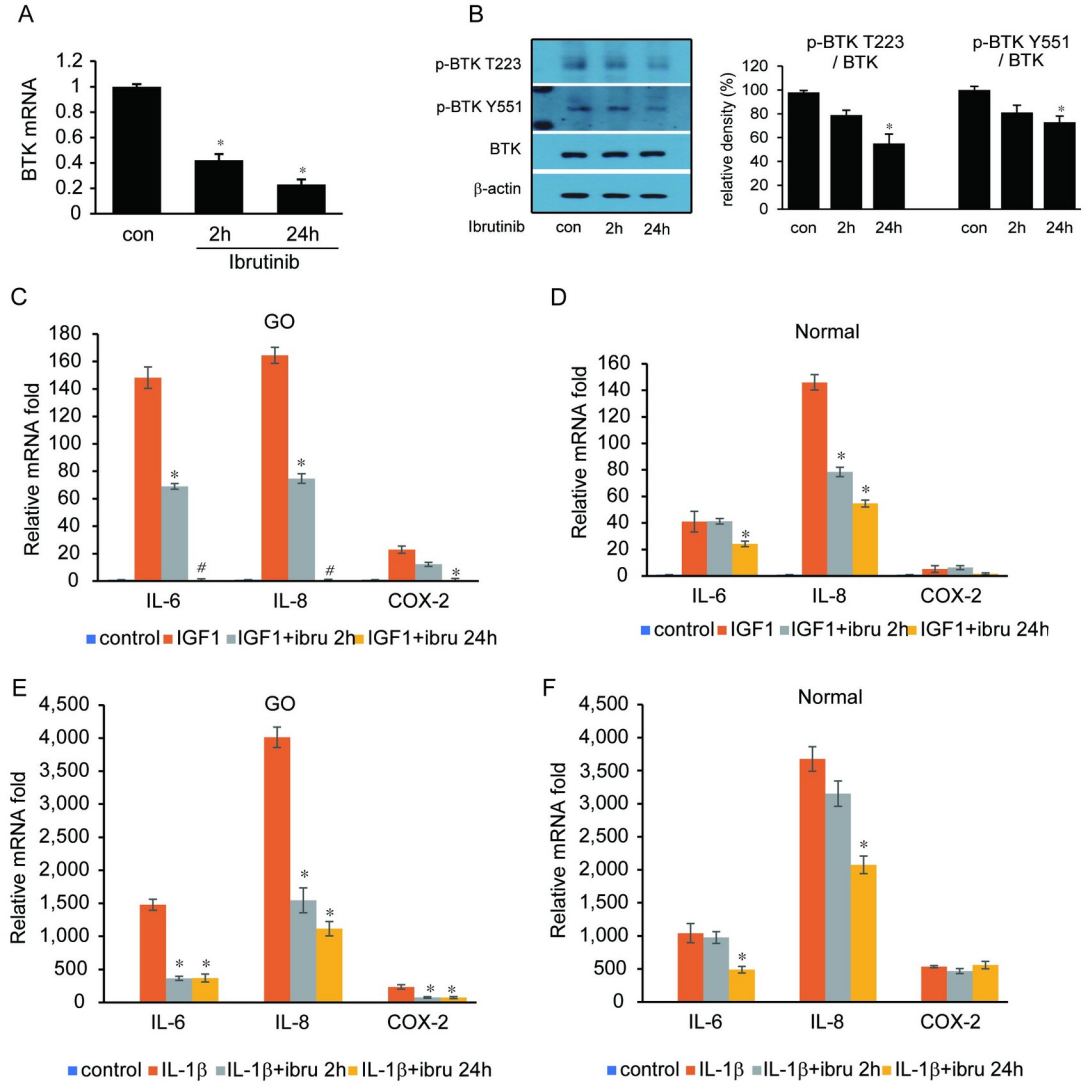
Effect of ibrutinib on proinflammatory cytokine production. (A) Orbital fibroblasts obtained from Graves’ orbitopathy (GO) patients were treated with ibrutinib (1 μM) for 2 and 24 hours. BTK gene expression was inhibited by ibrutinib treatment in real time PCR analysis. (B) Production of phosphorylated BTK protein at T223 and Y551 domain was suppressed by ibrutinib treatment for 24hours. (C–F) Orbital fibroblasts from GO and normal subjects were incubated with ibrutinib for 2 and 24 hours prior to 24 hours of IGF-1 (10ng/mL) and IL-1β (10 ng/mL) treatment. In GO fibroblasts, IL-6, IL-8, and COX-2 mRNA expression levels were increased by treatment of IGF-1 (C) and IL-1ß (E), which were significantly attenuated by ibrutinib treatment. In normal fibroblasts, increased secretion of IL-6 and IL-8 by IGF-1 (D) and IL-1β (F) treatment was inhibited by ibrutinib treatment (*p<0.05, **#***p*<0.01). Abbreviations: ibru = ibrutinib.

**Fig 5:**
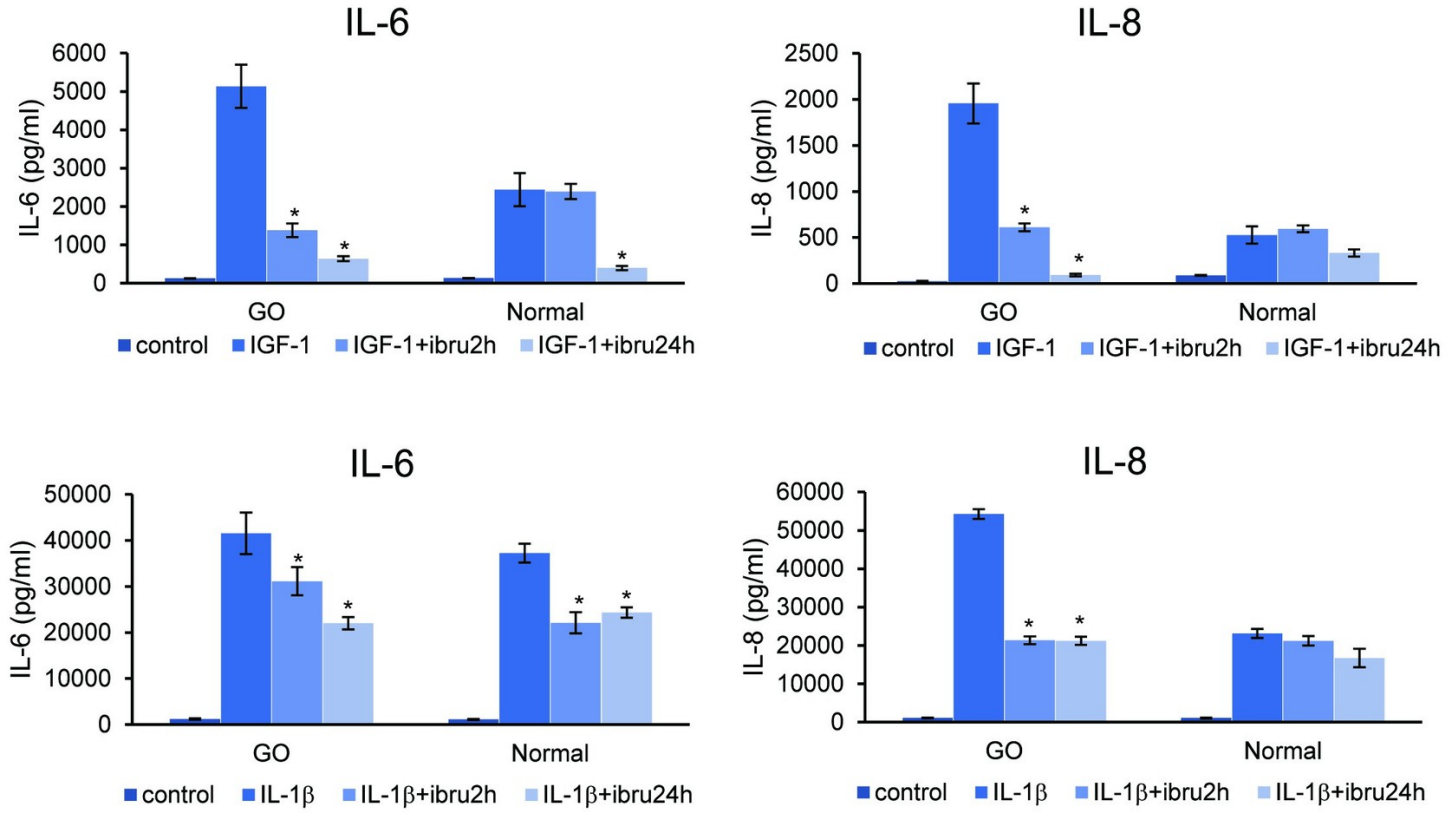
Effect of ibrutinib on IGF-1 and IL-1β induced proinflammatory cytokine protein secretion in Graves’ orbitopathy (GO) and normal orbital fibroblasts. Orbital fibroblasts from both GO and normal individuals were untreated or pretreated with ibrutinib (1 μM) for 2 and 24 hours prior to IGF-1 (10 ng/mL, 24 hours) or IL-1ß (10 ng/mL, 24 hours) treatment. The concentration of IL-6 and IL-8 were obtained using ELISA. The average value of three independent samples were presented and the results were shown as the means ± standard deviation (*p<0.05). Abbreviations: ibru = ibrutinib.

After confirming the suppressive effect of ibrutinib on expression of BTK transcription and phosphorylation in orbital fibroblasts (Fig 4A and 4B), the effect of ibrutinib on mRNA expression of proinflammatory cytokines was evaluated. First, RT-PCR results showed that stimulation with IGF-1 (10ng/mL) and IL-1β (10ng/mL) led to increased mRNA expression of the following proinflammatory cytokines: IL-6, IL-8, COX-2, and MCP-1 in both GO and normal orbital fibroblasts. In GO orbital fibroblasts, IGF-1 or IL-1β induced IL-6, IL-8 and COX-2 mRNA expression was significantly attenuated after ibrutinib (1 μM) treatment (Fig 4C and 4E). In normal orbital fibroblasts, IGF-1 or IL-1β induced IL-6 and IL-8 mRNA expression was suppressed by ibrutinib (Fig 4D and 4F). In the same experimental conditions, increased IL-6 and IL-8 protein secretion by IGF-1 or IL-1β stimulation was inhibited by ibrutinib treatment in GO cells. In normal control cells, only IL-6 production was suppressed by ibrutinib in both IGF-1 and IL-1ß induction (Fig 5). IL-1β induced MCP-1 secretion was also attenuated by ibrutinib treatment, whereas IGF-1 induced MCP-1 secretion was not affected by ibrutinib exposure (S2 Fig).

To assess the effect of ibrutinib on extracellular matrix synthesis, transcription levels of HAS1, HAS2, and HAS3 mRNA were evaluated by RT-PCR. In both GO and normal orbital fibroblasts, IL-1β (10ng/mL, 24hours) induced expression of all HAS mRNA was inhibited by ibrutinib (1 uM, 6 hours) treatment (S3 Fig).

### Effect of Ibrutinib on intracellular signaling pathways

**Fig 6:**
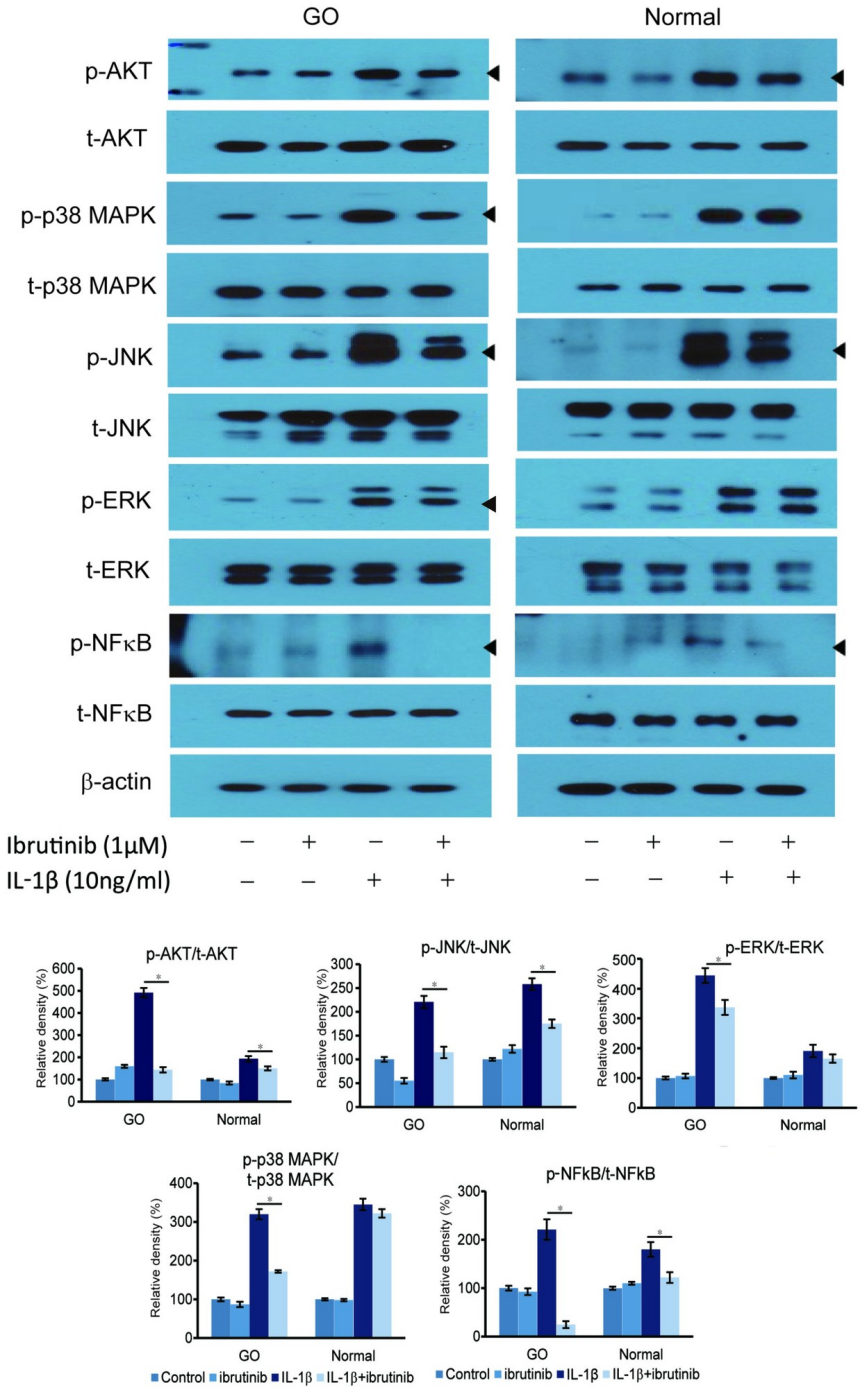
Effect of ibrutinib on intracellular signaling pathways in Graves’ orbitopathy (GO) and normal orbital fibroblasts. Fibroblasts were exposed to ibrutinib (1 μM) and then stimulated with IL-1β (10 ng/mL) for 1 hour. Cell lysates were analyzed by western blotting to investigate phosphorylated (p-) Akt, p38 MAPK, JNK, ERK, and NF-κB protein expression. Ibrutinib treatment significantly attenuated IL-1β-stimulated activation of p-Akt, p-JNK, and p-NF-κB in both GO and normal cells, whereas it suppressed activation of p-ERK and p-p38 MAPK in only GO cells. Each column in the graph represents a relative density ratio of each protein (p-level/total level) and were presented as means ± standard deviation. (*p<0.05 versus fibroblasts without ibrutinib treatment).

To determine signaling pathways affected by the regulation of BTK, western blot analyses were performed for multiple transcription factors. Cells were pre-treated with 1μM of ibrutinib for 1 hour and then stimulated with IL-1β (10 ng/mL) for 1 hour. Ibrutinib inhibited IL-1β-induced phosphorylation of Akt, JNK, and NF-κB in both GO fibroblasts and normal controls; p38 MAPK and ERK phosphorylation were suppressed in only GO fibroblasts (Fig 6).

## Discussion

Initially identified as a mutation in human immunodeficiency X-linked agammaglobulinemia, BTK has been implicated in inflammatory signaling, thereby holding promise as a therapeutic target for numerous autoimmune diseases [13, 14]. In this study, we investigated the role of BTK in the pathogenesis of GO by inducing inflammation using IL-1β and IGF-1 in primary cultured orbital fibroblasts. BTK was primarily overexpressed in GO tissue, compared to non-Graves’ normal orbital tissue, and exposure to both IGF-1 and IL-1β elicited increased level of BTK, including the phosphorylated form, in GO orbital fibroblasts. To our knowledge, this is the first study to demonstrate the overexpression of BTK in orbital tissue and fibroblasts from GO.

Recent studies reveal that BTK expression is implicated in numerous autoimmune diseases. BTK expression level in peripheral B-cells correlates with disease severity in a variety of autoimmune diseases including rheumatoid arthritis, primary Sjogren syndrome, lupus nephritis, IgA nephropathy, and active granulomatosis with polyangiitis [29–32]. BTK is mainly expressed in B-cell cytoplasm and plays crucial role in BCR signaling [33]. Upon BCR engagement, activated PI3K accumulates phosphatidylinositol (3,4,5) triphosphate (PIP3) and phosphorylation of spleen tyrosine kinase (SYK) leads to translocation and phosphorylation of BTK at Y551 domain, which is a catalytic domain. This leads to autophosphorylation at the T223 domain of BTK, and thereby activation of subsequent downstream signaling pathways and transcription factors such as NF-κB and several MAP kinases [34]. The NF-κB pathway is a major regulator in the inflammatory response, including the CD40-mediated B-cell and T-cell interaction, which is one of the main mechanisms of GO pathogenesis [35, 36]. We discovered that inhibition of BTK curtails phosphorylation of NF-κB and MAPK signaling in IL-1β treated orbital fibroblasts.

Numerous studies also suggest the role of BTK in innate immunity, such as in monocytes and dendritic cells. BTK can directly interact with Toll/IL-1 receptor domains of Toll-like receptors (TLR) 4, 6, 8, and 9 [17]. TLR4 signaling is believed to transduce signals through the MYD88 signaling pathway, leading to NF-κB activation, and also through the BCR signaling pathway, resulting in SYK, ERK, and Akt activation [37]. Rip J. et al. also suggested that BTK expression renders autoreactive B-cells more sensitive to TLR stimulation [38]. Furthermore, the TLR4, 9 and BCR interactions synergistically increase IL-6 production in primary murine B-cells [39]. Taken together, the BTK mediated BCR and TLR interactions may play a crucial role in autoimmune diseases. In the context of autoimmune thyroid disease, expression of TLR2 and TLR4 positive T-cells and B-cells were higher in the peripheral blood of Graves’s disease patients compared to normal controls, and the proportion significantly decreases after obtaining euthyroid status [40]. We also previously showed that TLR2 and TLR4 mRNA level are significantly higher in GO tissues than in normal tissues, and TLR2 inhibition significantly reduces proinflammatory cytokines including IL-6 and IL-8 [41]. There is a possibility that BTK activation might boost the B-cell mediated inflammatory response in GO via the TLR signaling pathways.

Ibrutinib, the first orally available BTK selective inhibitor, is approved for the treatment of various B-cell malignancies and is now being studied in pre-clinical and clinical trials examining its anti-inflammatory effect in autoimmune diseases [19, 26, 27, 42]. *In vitro* treatment of ibrutinib in peripheral blood from systemic sclerosis reduces IL-6 and TNF-α production, while IL-10 is preserved [42]. Ibrutinib treatment also attenuates NF-κB and NLRP3 inflammasome activation in metabolic inflammation in an *in vivo* murine model of diabetes mellitus [43]. Furthermore, treatment with ibrutinib abrogated pulmonary injury in COVID-19 infected individuals [44, 45]. In our study, pretreatment with ibrutinib significantly suppressed the production of IL-6, IL-8 and COX-2 in both IGF-1 and IL-1β stimulated GO orbital fibroblasts and MCP-1 production in IL-1β stimulated GO orbital fibroblasts, which are important inflammatory mediators in GO [35]. Ibrutinib treatment also curtailed phosphorylation of Akt, p38, JNK, ERK and NF-kB signaling protein in GO fibroblasts, which is known to be involved in GO pathogenesis [46]. Our study demonstrates targeting BTK by a selective BTK inhibitor may exert a therapeutic effect in the inflammatory mechanism of GO.

Excessive HA production and deposition in the orbital soft tissues is a major cause of orbital tissue expansion in GO [6]. After stimulation with proinflammatory cytokines such as IL-1β, orbital fibroblasts massively produce HA, and its synthesis is regulated by HAS1, 2, and 3 [47, 48]. These enzymes lengthen hyaluronan by repeatedly adding glucuronic acid and N-acetylglucosamine to the nascent polysaccharide. IL-1β strongly enhances HAS1, 2, and 3 protein expression in orbital fibroblasts, which is blocked by dexamethasone treatment [47]. Combined blockage of PI3K1A and mTORC1 signaling inhibited HAS2 transcription and hyaluronan accumulation in orbital fibroblasts [49]. In this study, ibrutinib suppressed all three types of IL-1β induced HAS mRNA expression in both GO and normal orbital fibroblasts. The underlying mechanism related to HAS gene suppression is not entirely clear, but our study revealed that ibrutinib attenuated IL-1β-induced Akt and MAPK activation, and this may have inhibited IL-1β induced HAS1,2 and 3 transcription.

The precise mechanism of anti-inflammatory response of ibrutinib in GO pathogenesis remains unclear and needs to be elucidated. Ibrutinib has off-target kinase inhibitory effects toward other Tec family kinases, especially IL-2-inducible T-cell kinase (ITK), which plays pivotal role in T-cell development [50, 51]. T-cells also infiltrate orbital tissues in GO, in addition to B-cells, providing the proinflammatory microenvironment [1, 2]. Combined inhibitors of the T-cell mediated response may have a higher yield in GO, considering the effect of both B-cells and T-cells in GO. Furthermore, after FDA approval, ibrutinib has been used in clinical settings in various B-cell malignancies, and the bioavailability and safety profile of ibrutinib has been verified. A number of BTK inhibitors with higher sensitivity are currently being developed to lessen adverse events and to reduce unwanted off-target activities [52, 53]. Further *in vivo* and clinical studies are necessary to investigate the actual effect of BTK targeting on GO pathogenesis and possible side effects.

In conclusion, we demonstrate that the inhibition of BTK has anti-inflammatory effects in GO fibroblasts in an *in vitro* model. Ibrutinib, a selective BTK inhibitor suppresses proinflammatory cytokine production and abrogates activation of multiple proinflammatory transcription factors. Taken together, our finding suggests the possibility that ibrutinib may be a promising therapeutic candidate in GO. Further studies are necessary to establish the clinical validation of BTK inhibitors in *in vivo* and clinical settings.

## Supporting information

S1 Fig Full length gels representative of Western blot analysis for Figs 2, 4 and 6. (TIF)

S2 Fig Effect of ibrutinib on MCP-1 mRNA expression. Orbital fibroblasts were untreated or pre-treated with ibrutinib (1 μM) for 2 or 24 hours and stimulated with IL-1β (10 ng/mL, 6 hours). In GO fibroblasts, both 2 and 24 hours of ibrutinib treatment significantly curtailed IL-1β induced MCP-1 expression, whereas IL-1β induced MCP-1 expression was only suppressed after 24 hours of ibrutinib exposure in normal orbital fibroblasts.
(TIF)

S3 Fig Effect of ibrutinib on mRNA expression levels of Hyaluronan synthase (HAS) 1, HAS 2, and HAS 3 in both GO and normal orbital fibroblasts. Orbital fibroblasts were untreated or incubated with ibrutinib (1 μM) for 24 hours and then stimulated with IL-1β (10 ng/mL, 6 hours). Transcription levels of HAS1, HAS2, and HAS3 were evaluated by using real-time quantitative PCR. Ibrutinib pre-treatment significantly hindered IL-1beta induced HAS1, HAS2, and HAS3 expression in both GO and normal orbital fibroblasts. (*p<0.05 versus fibroblasts without ibrutinib treatment).
(TIF)

S1 Table Demographics of the patients’ sample in this study. (DOCX)

S1 File (XLSX)
