# Supplementary figures and images for: Therapeutic effect of ibrutinib, a selective Bruton’s tyrosine kinase inhibitor, in orbital fibroblasts from patients with Graves’ orbitopathy
Source: PLoS One. 2022 Dec 15;17(12):e0279060. doi: 10.1371/journal.pone.0279060 (PMC9754806; doi:10.1371/journal.pone.0279060)

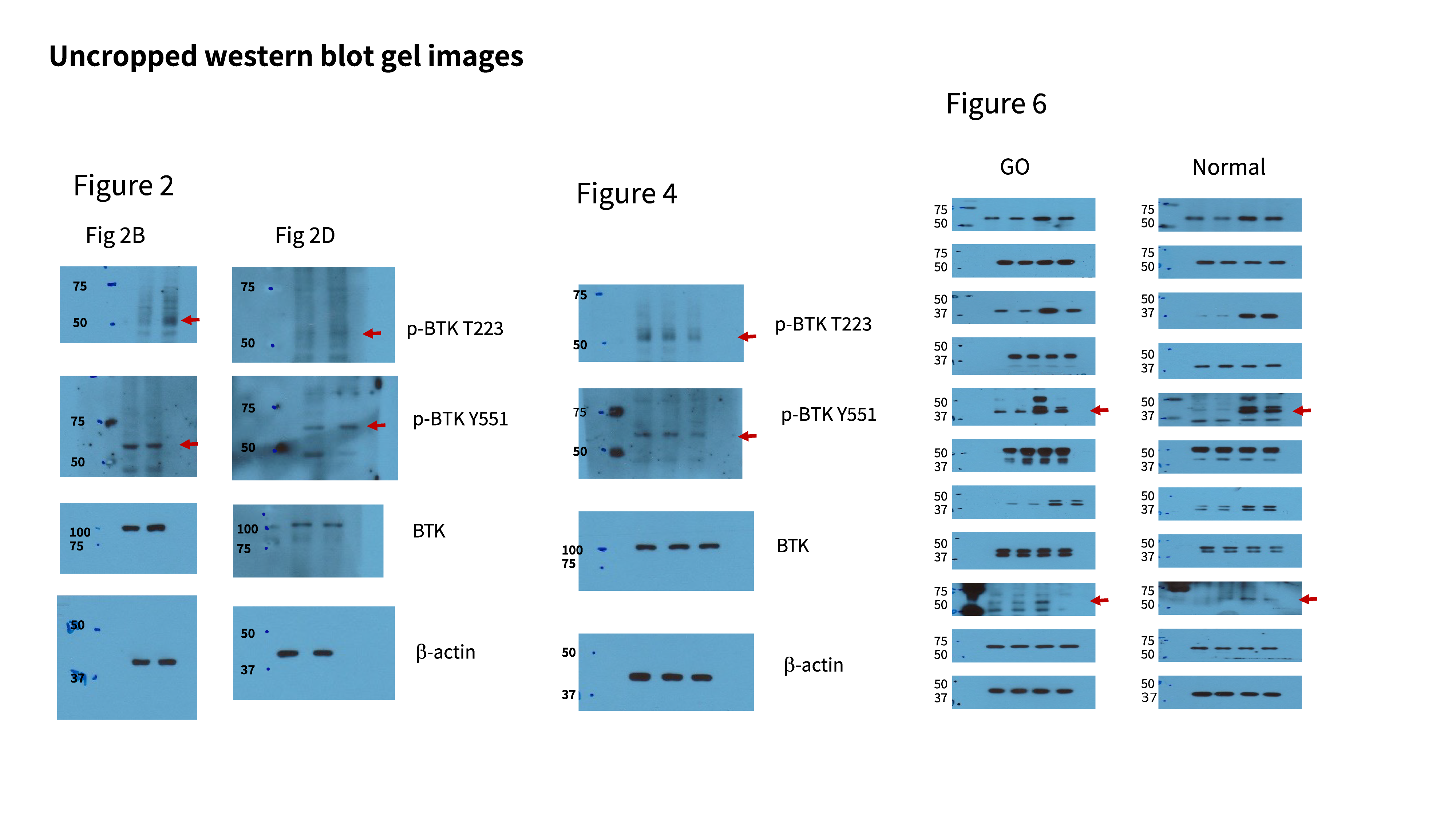

Supplement: S1 Fig — (TIF) [file pone.0279060.s001.tif]

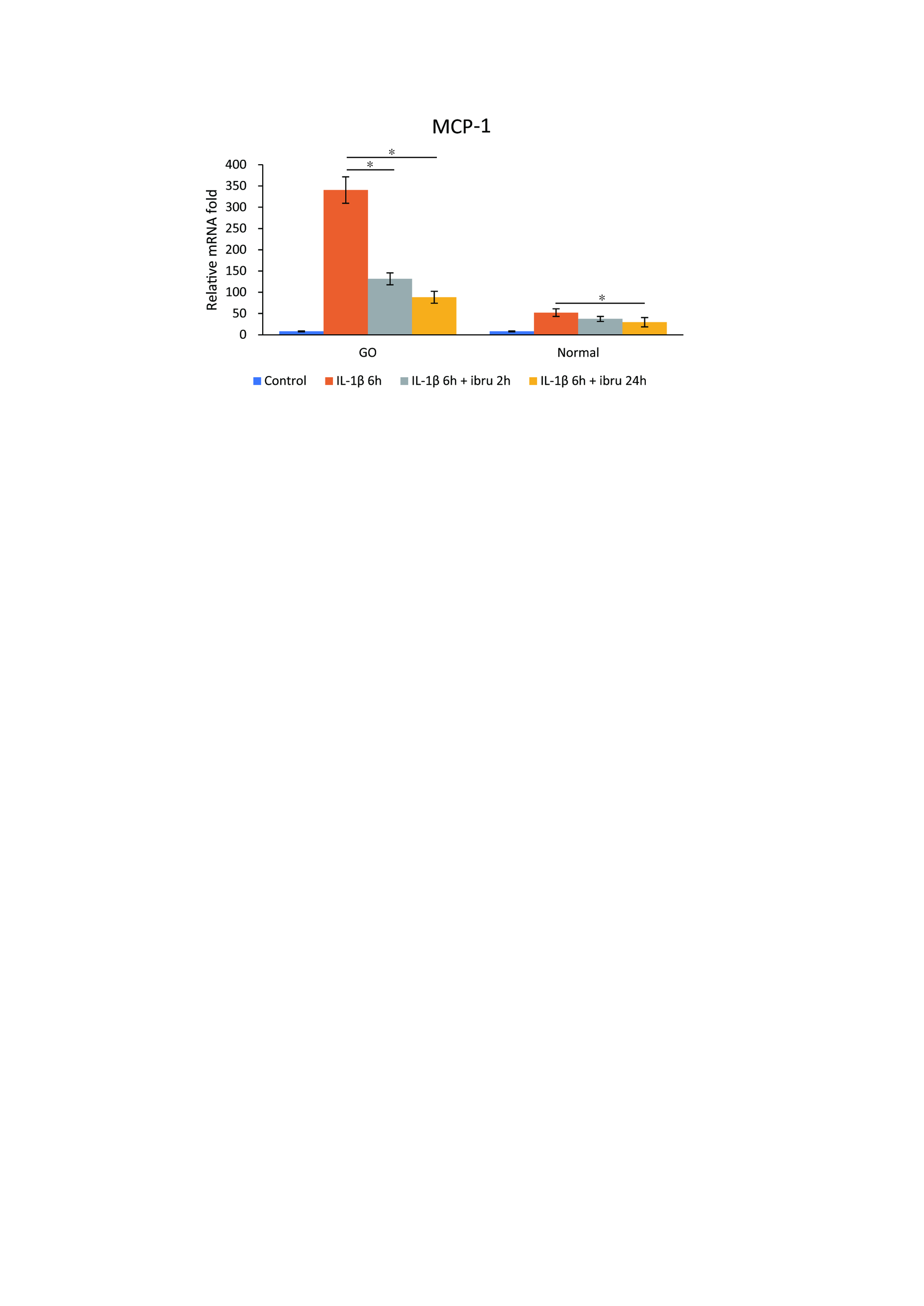

Supplement: S2 Fig — Orbital fibroblasts were untreated or pre-treated with ibrutinib (1 μM) for 2 or 24 hours and stimulated with IL-1β (10 ng/mL, 6 hours). In GO fibroblasts, both 2 and 24 hours of ibrutinib treatment significantly curtailed IL-1β induced MCP-1 expression, whereas IL-1β induced MCP-1 expression was only suppressed after 24 hours of ibrutinib exposure in normal orbital fibroblasts. (TIF) [file pone.0279060.s002.tif]

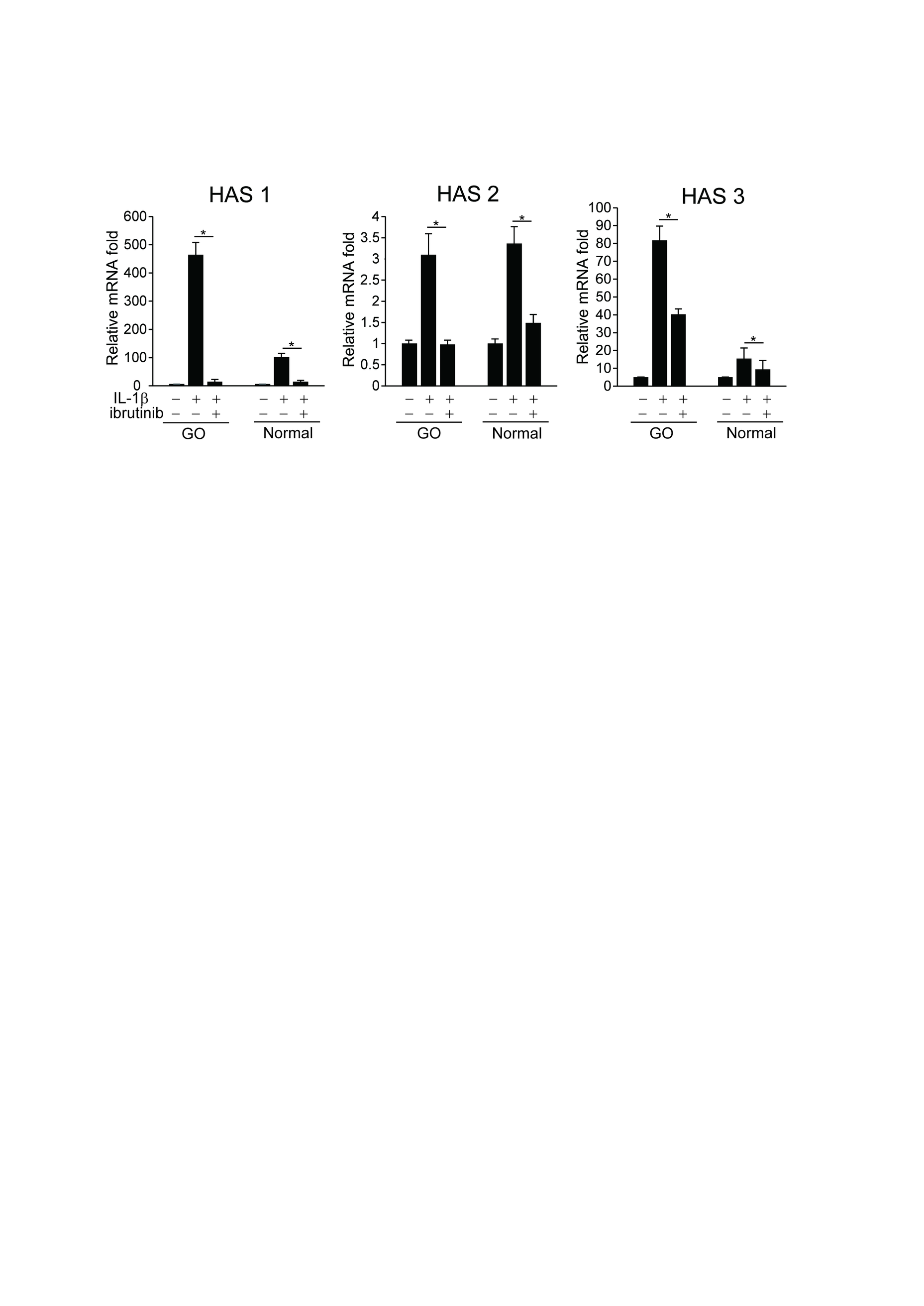

Supplement: S3 Fig — Orbital fibroblasts were untreated or incubated with ibrutinib (1 μM) for 24 hours and then stimulated with IL-1β (10 ng/mL, 6 hours). Transcription levels of HAS1, HAS2, and HAS3 were evaluated by using real-time quantitative PCR. Ibrutinib pre-treatment significantly hindered IL-1beta induced HAS1, HAS2, and HAS3 expression in both GO and normal orbital fibroblasts. (*p<0.05 versus fibroblasts without ibrutinib treatment). (TIF) [file pone.0279060.s003.tif]
