# Supplementary material for: Therapeutic effect of ibrutinib, a selective Bruton’s tyrosine kinase inhibitor, in orbital fibroblasts from patients with Graves’ orbitopathy
Source: PLoS One. 2022 Dec 15;17(12):e0279060. doi: 10.1371/journal.pone.0279060 (PMC9754806; doi:10.1371/journal.pone.0279060)
Supplement: S1 Table — (DOCX) [file pone.0279060.s004.docx]

**Supplementary table 1. Demographics of the patients’ sample in this study**

| No | Age | Sex | Duration(year) | CAS | Proptosis | Surgery |
| --- | --- | --- | --- | --- | --- | --- |
| Graves’ orbitopathy | | | | | | |
| 1 | 25 | F | 1.5 | 0/7 | 20/20 | Decompression |
| 2 | 28 | F | 1.3 | 0/7 | 21/21 | Decompression |
| 3 | 24 | F | 3.5 | 0/7 | 19/20 | Decompression |
| 4 | 36 | F | 6.5 | 3/7 | 21/21 | Decompression |
| 5 | 33 | M | 11.0 | 2/7 | 20/20 | Decompression |
| 6 | 44 | F | 2.5 | 2/7 | 20/20 | Decompression |
| 7 | 40 | F | 1.5 | 3/7 | 18.5/14.5 | Decompression |
| 8 | 46 | M | 2.5 | 3/7 | 26/26 | Decompression |
| 9 | 37 | F | 7.5 | 2/7 | 26/26 | Decompression |
| 10 | 29 | F | 6.1 | 3/7 | 20.5/21.5 | Decompression |
| 11 | 41 | F | 1.6 | 0/7 | 21/21 | Decompression |
| 12 | 35 | F | 3.6 | 1/7 | 23/23 | Decompression |
| 13 | 49 | F | 4.1 | 2/7 | 23/21 | Decompression |
| 14 | 58 | M | 3.9 | 1/7 | 20.5/22 | Decompression |
| 15 | 62 | F | 1.3 | 2/7 | 21/20 | Decompression |
| Normal healthy control | | | | | | |
| 1 | 43 | F | NA | NA | NA | Upper blepharoplasty |
| 2 | 69 | M | NA | NA | NA | Upper blepharoplasty |
| 3 | 61 | F | NA | NA | NA | Upper blepharoplasty |
| 4 | 44 | F | NA | NA | NA | Upper blepharoplasty |
| 5 | 67 | M | NA | NA | NA | Lower blepharoplasty |
| 6 | 25 | F | NA | NA | NA | Upper blepharoplasty |
| 7 | 42 | F | NA | NA | NA | Upper blepharoplasty |
| 8 | 24 | M | NA | NA | NA | Upper blepharoplasty |
| 9 | 41 | F | NA | NA | NA | Lower blepharoplasty |
| 10 | 29 | F | NA | NA | NA | Lower blepharoplasty |
| 11 | 33 | F | NA | NA | NA | Lower blepharoplasty |
| 12 | 41 | F | NA | NA | NA | Lower blepharoplasty |
| 13 | 46 | M | NA | NA | NA | Upper blepharoplasty |
| 14 | 51 | F | NA | NA | NA | Upper blepharoplasty |

Abbreviations: CAS, clinical activity scores; F, female; M, male; NA, not applicable.
